# Clonality and α-a Recombination in the Australian *Cryptococcus gattii* VGII Population - An Emerging Outbreak in Australia

**DOI:** 10.1371/journal.pone.0016936

**Published:** 2011-02-24

**Authors:** Fabian Carriconde, Félix Gilgado, Ian Arthur, David Ellis, Richard Malik, Nathalie van de Wiele, Vincent Robert, Bart J. Currie, Wieland Meyer

**Affiliations:** 1 Molecular Mycology Research Laboratory, Sydney Medical School - Westmead Hospital, Centre for Infectious Diseases and Microbiology, Westmead Hospital, Westmead Millennium Institute, Sydney Emerging Infections and Biosecurity Institute, The University of Sydney, Sydney, New South Wales, Australia; 2 PathWest Laboratory Medicine WA, QEII Medical Centre, Nedlands, Western Australia, Australia; 3 SA Pathology at Women's and Children's Hospital, North Adelaide, South Australia, Australia; 4 Centre for Veterinary Education, The University of Sydney, Sydney, New South Wales, Australia; 5 Hogeschool, Leiden, The Netherlands; 6 CBS-Fungal Biodiversity Center, Utrecht, The Netherlands; 7 Tropical and Emerging Infectious Diseases Division, Menzies School of Health Research, Northern Territory Clinical School and Infectious Diseases Department, Royal Darwin Hospital, Casuarina, Northern Territory, Australia; University of Minnesota, United States of America

## Abstract

**Background:**

*Cryptococcus gattii* is a basidiomycetous yeast that causes life-threatening disease in humans and animals. Within *C. gattii*, four molecular types are recognized (VGI to VGIV). The Australian VGII population has been in the spotlight since 2005, when it was suggested as the possible origin for the ongoing outbreak at Vancouver Island (British Columbia, Canada), with same-sex mating being suggested as the driving force behind the emergence of this outbreak, and is nowadays hypothesized as a widespread phenomenon in *C. gattii*. However, an in-depth characterization of the Australian VGII population is still lacking. The present work aimed to define the genetic variability within the Australian VGII population and determine processes shaping its population structure.

**Methodology/Principal Findings:**

A total of 54 clinical, veterinary and environmental VGII isolates from different parts of the Australian continent were studied. To place the Australian population in a global context, 17 isolates from North America, Europe, Asia and South America were included. Genetic variability was assessed using the newly adopted international consensus multi-locus sequence typing (MLST) scheme, including seven genetic loci: *CAP59, GPD1, LAC1, PLB1, SOD1, URA5* and IGS1. Despite the overall clonality observed, the presence of *MAT* **a** VGII isolates in Australia was demonstrated for the first time in association with recombination in *MATα*-*MAT* ***a*** populations. Our results also support the hypothesis of a “smouldering” outbreak throughout the Australian continent, involving a limited number of VGII genotypes, which is possibly caused by a founder effect followed by a clonal expansion.

**Conclusions/Significance:**

The detection of sexual recombination in *MATα*-*MAT* ***a*** population in Australia is in accordance with the natural life cycle of *C. gattii* involving opposite mating types and presents an alternative to the same-sex mating strategy suggested elsewhere. The potential for an Australian wide outbreak highlights the crucial issue to develop active surveillance procedures.

## Introduction

Life-threatening infections due to fungi have increased significantly over recent decades, posing new challenges for public health [1]–[3]. Fungal emergence appears to be driven by various factors, including rising numbers of immunocompromised patients and the development of antimicrobial resistance [2], [4]. In the context of a worldwide expansion of fungal pathogens, it is essential to understand the taxonomy, epidemiology, ecology and population biology of the fungi involved.

The two basidiomycetous haploid yeasts, *Cryptococcus neoformans* and *Cryptococcus gattii* are causative agents of cryptococcosis, a serious disease that manifests as meningitis and meningoencephalitis in humans [5]. *C. neoformans* has a worldwide distribution and infects predominantly patients with impaired immunity. In contrast, *C. gattii* infection has been mostly associated with immunocompetent hosts and was originally designated as a tropical and subtropical pathogen [6]. Infections due to *C. gattii* have been reported from human and a wide range of animal species [7]–[9]. Cryptococcosis is initially caused by the inhalation of airborne infectious propagules released from environmental niches. Because of the well-known behaviour of aerosolized particles after inhalation it is presumed that suitable inocula are either basidiospores or desiccated yeast cells [10]. Determination of the primary ecological niches of *C. gattii* is of great importance to better understand its life cycle and thereby determine exposure risks and implement preventive strategies, as required.

In Australia, numerous studies have revealed an association between *C. gattii* VGI and eucalyptus trees, particularly with the native species *Eucalyptus camaldulensis* (river red gum) [11]. Viable yeast cells have been commonly isolated from woody debris and detritus in hollows of mature trees and sometimes in nearby soil [11]–[13]. It was initially postulated that exposure to eucalyptus trees may account for the high incidence of cryptococcosis within Australia [10]. Subsequently, associations between *C. gattii* and trees have been reported from other host plants in various countries [7], [12], [14], [15], indicating the existence of additional ecological niches. Nevertheless, the high prevalence of *C. gattii* in Australia and its association with native eucalypt trees and the extensive exportation of those trees led to the hypothesis that *C. gattii* originated from Australia and was subsequently dispersed into other parts of the world through man-made horticulture [10], [16], [17].

Besides studying the environmental niche of a potential human pathogen, the determination of the relative importance of sexual *versus* asexual reproductions in the life cycle of a fungus is a crucial biological issue. Sex allows for new genetic recombination and increases the potential for adaptation to new environments [18]. Furthermore, it potentially results in the emergence of new virulent genotypes [19]. On the other hand, asexual reproduction enables the propagation of well-adapted clones to certain environmental conditions without disrupting favourable gene combinations [20]. *Cryptococcus* can reproduce both sexually and asexually. Sexual reproduction involves a bipolar mating system with two mating type alleles, *MAT* **a** and *MAT*α. Mating occurs between opposite mating types, resulting in the formation of basidiospores. Asexual reproduction occurs via budding [10]. Recently, same sex mating between two α cells has been suggested to occur naturally in *C. gattii* [16]. However, for its sibling species *C. neoformans*, this has only been observed under laboratory conditions [21].

Within *C. gattii*, four molecular types are recognized: VGI, VGII, VGIII and VGIV [22], which may in fact represent different varieties or phylogenetic species [23]. VGI is the major molecular type recovered from clinical, veterinary and environmental samples from eastern Australia where human populations are most concentrated [24]. In addition, numerous VGII infections have been reported in Australia from the eastern states, from the southwest of Western Australia (WA) and the Northern Territory [25], [26]. The Australian VGII population has been in the spotlight since 2005, when it was suggested as the possible origin of an on-going outbreak of cryptococcosis at Vancouver Island, BC, Canada [16]. Two genotypes have been delineated as the causative agents of this outbreak, the major genotype VGIIa and the minor genotype VGIIb [16], [27]. Based on the fact that some Australian isolates had an identical genotype to VGIIb, which might represent a potential parental strain for the highly virulent VGIIa genotype, it was postulated that this genotype originated from Australia and subsequently was dispersed to the North Pacific coast. The association between eucalyptus trees and *C. gattii*, in concert with the large-scale exportation of these trees to other parts of the world over the last century supports the notion of an Australian origin for this fungus [10], [16]. Despite extensive environmental sampling only α mating type isolates have been observed from Vancouver Island, leading to the suggestion that same-sex mating between two α cells is the driving force for the emergence of the outbreak. Previous population genetic studies carried out on VGII populations from two Australian regions, the Northern Territory and the greater Sydney area have detected statistical evidence of recombination only when tests were performed between genetically closely related isolates, in the absence of any *MAT* **a** VGII isolates [17], [25], [28]. This finding supports the same-sex mating hypothesis [16].

To shed further light on the low virulent VGIIb Vancouver Island outbreak strain and its relationship with Australian isolates, the current study focused on (i) characterizing the genetic variability within the Australian *C. gattii* VGII population on a large geographical scale investigating 54 clinical, veterinary and environmental isolates from Queensland (QLD), New South Wales (NSW), Northern Territory (NT) and Western Australia (WA), using multilocus sequence typing (MLST); and (ii) determining the processes shaping its population structure, in particular the reproductive modes (sexual *vs* asexual).

## Results

### Genetic variability

The 7 sequenced loci (*CAP59, GPD1, LAC1, PLB1, SOD1, URA5* and IGS1) of the *Cryptococcus* consensus multilocus sequence typing (MLST) scheme adopted by the International Society of Human and Animal Mycology (ISHAM), resulted in 4166 bp nucleotide positions when aligned with the two reference strains of the Vancouver Island outbreak, CDC R265 (VGIIa) and CDC R272 (VGIIb), from which 47 polymorphic sites were identified (Table 1). When only Australian isolates were considered, 4165 bp were in the total aligned and 46 polymorphic sites were observed. This difference was due to the strain CDC R265 (VGIIa) presenting one additional nucleotide polymorphism compared to the Australian dataset at position 318 in the *GPD1* locus (Table 1). Thus, regarding the Australian population, among the 7 MLST loci studied, the number of polymorphic sites ranged from 12 for *SOD1* to 3 for *GPD1* and *URA5* (Table 1 and Table 2). From these polymorphisms, the highest number of alleles was observed for IGS1 (6 alleles), followed by *CAP59*, *PLB1* and *SOD1* (5 alleles each), *GPD1* and *LAC1* (4 alleles each) and *URA5* (3 alleles) (Table 2).

**Table 1:** Nucleotide polymorphism of the seven MLST loci (*CAP59*, *GPD1*, *LAC1*, *PLB1*, *SOD1*, *URA5* and IGS1) for the six sequence types (STs) delineated in this study.

MLST Locus		*CAP59*					*GPD1*				*LAC1*				*PLB1*								*SOD1*												*URA5*			*IGS1*										
	Position (bp)	7	79	220	424	547	86	106	318	330	283	371	406		63	157	168	277	382	484	486	511	35	97	211	387	396	430	435	464	496	527	536	550	144	146	263	37	127	294	351	376	412	429	481	542	597	604
Sequence Type																																																
**ST7 (n = 39)**		C T G C A					A T G T				C C C G				A G T C A A G A								A T G C G T C G G C A A												G C A			A G A A G G T T C T T										
**ST5 (n = 1)**		. C A . .					. C . .				. . . .				. . . . . . . G								G C C . . . T A A . . .												. . .			. . G . . . . . T . .										
**ST38 (n = 2)**		. . . . G					. . . .				. . . A				. . . . . . C G								G C . . T C T . A A G G												. . .			. . . . . A . . T C .										
**ST21 (n = 1)**		. C . T .					T . . .				. . G .				G G C T C G . G								G C . . T C T . A A G G												. T .			. . . C . . . . T C .										
**ST33 (n = 5)**		. C A . .					. . . A				T T . .				G C . T C G . G								G C . T . . T . A . . .												T . G			G T . . C . A G T . C										
**ST48 (n = 6)**		T C . . .					. . . .				. . . .				G C . T C G . G								G C . T . . . . A . . .												. T .			. . . . . . . . T . .										
**VGIIa = ST20**		. C . T .					. C A .				. . . .				G C . T C G . G								G . . T . . . . A . . .												. T .			. . . . . . . . T . .										
**VGIIb = ST7**		. . . . .					. . . .				. . . .				. . . . . . . .								. . . . . . . . . . . .												. . .			. . . . . . . . . . .										

Positions of the polymorphic nucleotides have been determined after alignment with the two reference strains CDC R265 and CDC R272 corresponding to VGIIa and VGIIb genotypes, respectively. Number of strains belonging to each sequence type is indicated between brackets.

**Table 2:** Sequence polymorphism summary of the 7 MLST loci used in this study for the 54 Australian isolates.

Locus	No. of aligned bases (bp)	No. of polymorphic sites	Number of alleles defined
***CAP59***	557^a^^,^ b	5^a^^,^ b	5^a^^,^ b
***GPD1***	549^a^; 548^b^	4^a^; 3^b^	5^a^; 4^b^
***LAC1***	475^a^^,^ b	4^a^^,^ b	4^a^^,^ b
***PLB1***	534^a^^,^ b	8^a^^,^ b	5^a^^,^ b
***SOD1***	712^a^^,^ b	12^a^^,^ b	5^a^^,^ b
***URA5***	638^a^^,^ b	3^a^^,^ b	3^a^^,^ b
***IGS1***	701^a^^,^ b	11^a^^,^ b	6^a^^,^ b
**Total**	4166^a^; 4165^b^	47^a^; 46^b^	33^a^; 32^b^

a Values when CDC R265 (VGIIa  =  ST20) was included.

b Values without CDC R265 sequences (VGIIa  =  ST20).

**Figure 1:**
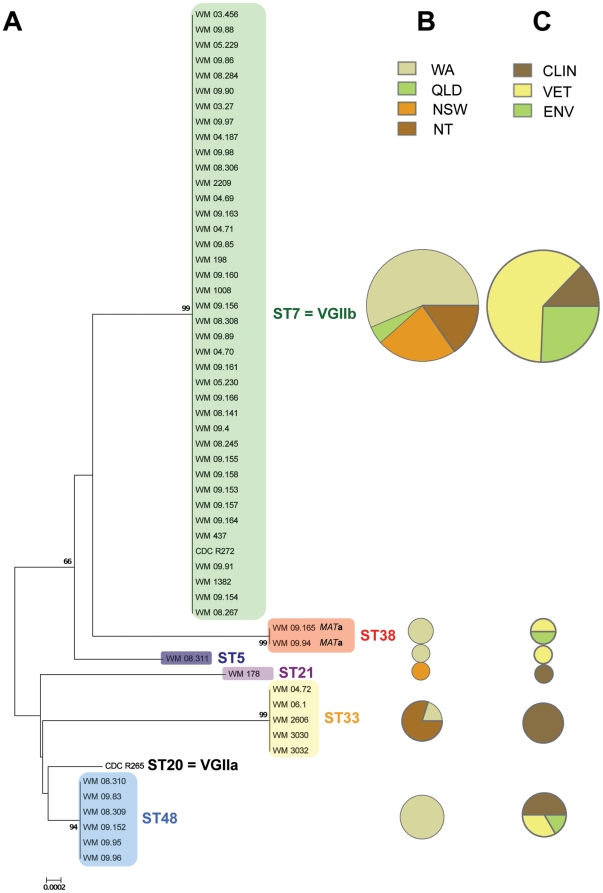
(A) Unrooted Neighbor-Joining consensus tree of the 54 *Crytococcus gattii* VGII Australian isolates and the two reference strains CDC R265 (VGIIa  =  ST20) and CDC R272 (VGIIb  =  ST7) based on the 7 concatenated MLST loci. The two ST38 isolates are *MAT*a, all others are *MATα*. For each sequence type the isolate proportions regarding their (**B**) geographical origin (QLD: Queensland, NSW: New South Wales, NT: Northern Territory and WA: Western Australia) and their (**C**) source of isolation (CLIN: clinical, VET: veterinary and ENV: environmental) are presented. Pie charts are proportional to the sampling size.

The allele combinations (Table S1) and the phylogenetic relationships (Figure 1) revealed six distinct sequence types among the 54 Australian isolates: ST5, ST7, ST21, ST33, ST38 and ST48. Thirty-nine isolates belonged to ST7, six to ST48, five to ST33, two to ST38 and one each to ST5 and ST21.

The majority of the Australian isolates (∼72%) belonged to a single sequence type (ST7), which was identical to the allelic profile of the reference strain CDC R272 corresponding to the VGIIb low virulent genotype from Vancouver Island (British Columbia, Canada) (Table 1 and Table S1). None of the Australian isolates had an allelic profile corresponding with the reference strain CDC R265 of the high virulent VGIIa genotype of the Vancouver Island outbreak. A different sequence type number was thus given to this strain - ST20 (Table S1 and Figure 1). The ST48 was the most closely related genotype to ST20 (Figure 1), with only 5 nucleotides differences over the 7 investigated loci (Table 1).

**Figure 2:**
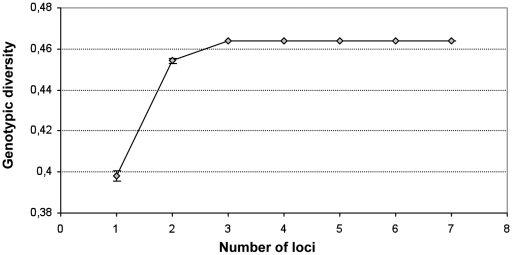
Relationships between the number of loci and the genotypic diversity in the Australian *C. gattii* VGII population. Each data point corresponds to the mean genotypic diversity and its standard error from 1000 permutations.

To determine whether scoring more loci would or wouldn't have increased the genetic diversity, the detected genotypic diversity was plotted against the number of loci analysed (Figure 2). This analysis clearly revealed that the genotypic diversity reached a plateau at 3 loci. Thus, the 7 loci used were sufficient to discriminate all observed sequence types within the Australian VGII population.

**Figure 3:**
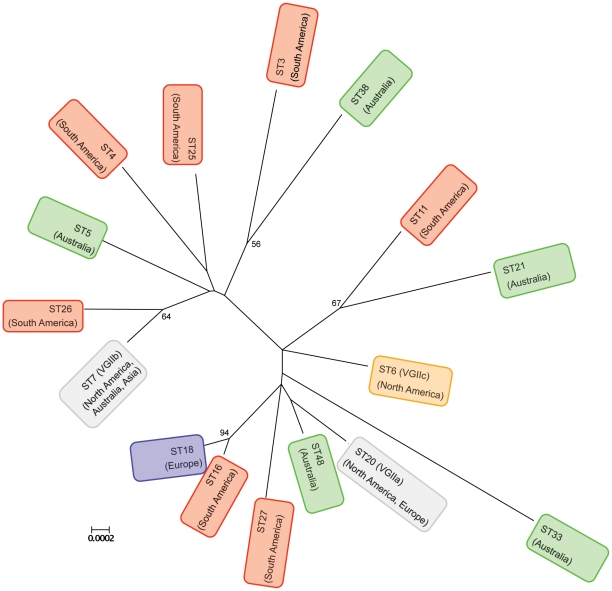
Unrooted Neighbor-Joining tree of the sequence types delineated for *C. gattii* VGII in this study and originated from different parts of the world. Bootstrap values over 50% are given at the nodes.

To place the Australian population in a global context additional isolates from North America, Europe, Asia and South America were studied (Table S1 and Figure 3). This analysis reemphasised the low genetic diversity found in the Australian VGII population. The highest genetic diversity within the global VGII population was seen in South American isolates, as shown by representative isolates (Table S1 and Figure 3) selected from an ongoing global VGII MLST study.

### Repartition of the genetic variability

**Figure 4:**
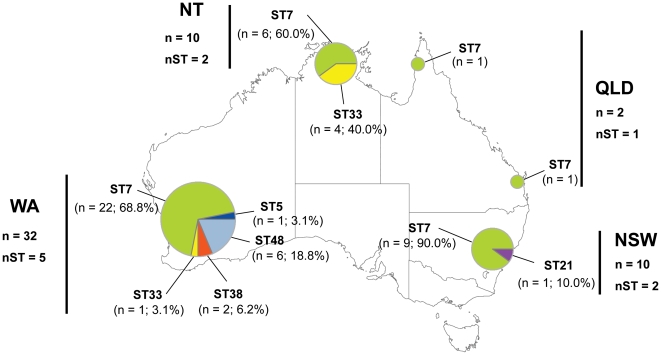
Spatial distribution of the different sequence types delineated in the Australian *C. gattii* VGII population. Pie charts are proportional to the number of samples. The symbols n and nST corresponds to the number of samples and the number of sequence types observed, respectively.

Looking at the geographical regions within Australia, WA showed the highest genetic diversity, with five sequence types detected out of the six present throughout Australia (Figure 4). Among the five genotypes detected in this state three were so far unique to WA (ST5, ST38 and ST48) (Figure 1 and Figure 4). In the NT and NSW, two sequence types were observed, while only one was delineated in QLD.

The ST7 was present in all regions investigated and was by far the most common sequence type (Figure 1 and Figure 4). Indeed, in NSW and WA, 90.0% (n = 9) and 68.8% (n = 22) of the isolates, respectively, belonged to this ubiquitous MLST type. Likewise 2/2 isolates genotyped in QLD were identified as ST7. In the NT, ST7 was also the main sequence type (60.0%; n = 6), with ST33 being the only other sequence type detected (40.0%; n = 4). The ST33 genotype appeared to have a large distribution, being present in the NT and WA. However, it was found only once in WA. In NSW, in addition to ST7, a sequence type unique to this region was delineated (ST21).

**Figure 5:**
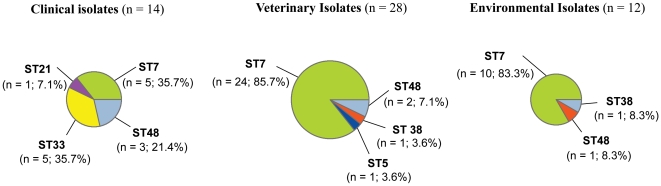
Repartition of the sequence types according to their source of isolation.

The distribution of the genetic diversity was also investigated in relation to the source of isolation (Figure 5). Amongst the human clinical isolates, four sequence types were delineated: ST7 (n = 5; 35.7%), ST21 (n = 1; 7.1%), ST33 (n = 5; 35.7%) and ST48 (n = 3; 21.4%). Isolates from veterinary cases were distributed among four distinct MLST types: ST5 (n = 1; 3.6%), ST7 (n = 24; 85.7%), ST38 (n = 1; 3.6%) and ST48 (n = 2; 7.1%). From environmental isolates, three sequence types were observed, ST7 (n = 10; 83.3%), ST38 (n = 1; 8.3%) and ST48 (n = 1; 8.3%). ST7 and ST48 were therefore obtained from all three sources of isolation (clinical, veterinary and environmental) (Figure 1 and Figure 5). Finally, three genotypes present in the environment were also recovered from animals (ST7, ST38 and ST48). In contrast, ST21 and especially ST33 were only obtained from human clinical isolates and ST5 was only isolated from a dog with cryptococcosis (Table S1). The lack of these sequence types from environmental samples highlights the need for further extensive sampling of *C. gattii* VGII isolates in Australia.

### Mating types and multilocus linkage disequilibrium

Mating type PCR revealed that 52 out of the 54 *C. gattii* VGII isolates studied were mating type α (Table S1). The remaining two isolates were of mating type **a**: WM 09.165 and WM 09.94 (Table S1). Both isolates belonged to ST38 and originated from WA (Table S1 and Figure 1). Isolate WM 09.94 was obtained from a 3-year-old dog from Geraldton with meningitis, while the isolate WM09.165 was recovered from a eucalyptus tree trunk (species not determined) at Caversham Wildlife Park (13 km from Perth).

In order to test for linkage disequilibrium among the seven loci and consequently investigate the presence of recombination, the *I_A_* and *rBard* association indexes were calculated. Both indexes were computed on the complete and clone-corrected datasets for populations having more than 3 sequence types, thus, for the overall Australian and the restricted WA populations. The clone-corrected analysis was performed by removing replicates of the same sequence type, as repetition of the same sequence type, due to clonality, can lead to the detection of linkage disequilibrium and consequently could affect the ability to detect recombination among genotypes. Indeed, when all isolates were included in the analyses, both the *I_A_* and *rBard* tests strongly rejected the null hypothesis of no linkage disequilibrium, which would indicate the absence of recombination (Table 3). However, after clone correction, the null hypothesis was not rejected (Table 3), indicating the absence of linkage disequilibrium and therefore suggesting the potential existence of recombination. Furthermore, the presence of α and **a** mating type isolates in these populations suggests that recombination may be occurring between the two opposite mating types.

**Table 3:** Multilocus linkage disequilibrium analyses performed on *C. gattii* VGII Australian populations.

Population (n)	*I_A_* (*p*-value)	*rBarD* (*p*-value)
***Australian population***		
All samples (54)	4.683 (<0.001)	0.782 (<0.001)
Clone-corrected (6)	−0.098 (0.725)	−0.027 (0.725)
***Western Australian population***		
All samples (32)	4.070 (<0.001)	0.685 (<0.001)
Clone-corrected (5)	−0.290 (0.863)	−0.077 (0.863)

### Demographic history

**Figure 6:**
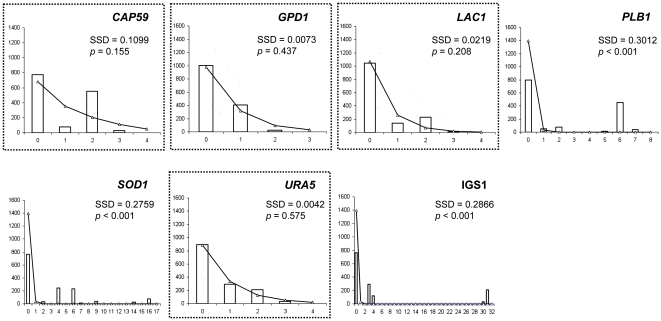
Observed (white bars) and expected (solid line and triangles) mismatch distributions under the sudden expansion model for the seven loci used in this study. The abscissa corresponds to the number of nucleotide differences between pairwise of sequences and the ordinate to the frequency. Pairwise nucleotide differences were realized on the global *C. gattii* VGII Australian population. Dashed lines represent the 90% confident interval of the expected mismatch distribution. Goodness-of-fit between the observed and expected mismatch distributions were tested using the sum of square deviation index (SSD) and loci for which a good match has been detected are highlighted by dashed squares.

The historical demography of the Australian *C. gattii* VGII population was investigated by analysing the pairwise sequence differences via mismatch distribution and neutrality tests. Mismatch distributions for four of the seven loci were adjusted to the distribution predicted under the sudden expansion model and were L-shaped (Figure 6). The genetic loci *CAP59*, *GPD1*, *LAC1*, and *URA5* showed no significant differences between observed and expected mismatch distributions (SSD *p*-values>0.05), and overall high pairwise frequency comparisons were obtained from 0 to 2 nucleotide differences. This goodness-of-fit between observed and expected pairwise difference distributions is likely to indicate an historical population expansion. The three neutrality tests, Tajima's *D*, Fu & Li's F* and Fu's *Fs*, failed to reveal a departure from the null hypothesis of neutral selection and/or population at equilibrium for all MLST loci (Table 4). However, slightly negative values were observed, which are expected when there is an excess of singletons (substitutions present in only one sampled sequence). The lack of significant values could be due to the large number of isolates belonging to the same sequence type, leading to high frequencies of 0 pairwise differences.

**Table 4:** Neutrality tests (Tajima's *D*, Fu & Li's F* and Fu's *F_S_*) performed on the seven MLST loci. None of the statistics gave significant *p*-values.

Locus	Tajima's *D*	Fu & Li's F*	Fu's *Fs*
***CAP59***	−0.462	−0.077	−0.381
***GPD1***	−1.274	−1.873	−2.446
***LAC1***	−1.082	−0.486	−1.012
***PLB1***	0.803	0.761	2.751
***SOD1***	−0.205	0.214	3.079
***URA5***	−0.265	0.624	0.715
**IGS1**	−0.903	−0.144	0.600

## Discussion

Life-threatening fungal infections represent a major contemporary challenge owing to their increasing occurrence and the emergence and re-emergence of outbreaks [2], [16], [29], [30]. It is therefore critical to investigate the population biology and the epidemiology of these organisms in order to better understand the associated risks of expansion to new environments, where indigenous human and animal populations are immunologically naïve and therefore at increased risk of infection. Indeed, the epidemiology of disease is closely related to the life history of fungi, including their reproductive strategies and dispersal abilities. In nature, there is a large continuum of breeding behaviour, ranging from exclusively asexual to fully sexual organisms [31].

The present study revealed a relatively low genetic diversity within the Australian *C. gattii* VGII population, with only six MLST types delineated amongst the 54 clinical, veterinary and environmental isolates studied. The majority (∼72%) of all isolates belonged to a single widely distributed sequence type, namely ST7, corresponding to VGIIb, the minor/less virulent genotype involved in the Vancouver Island outbreak [16], [27]. The over-representation of one sequence type suggests a clonal structure for the Australian *C. gattii* VGII population. Among the 75 *C. gattii* VGII isolates typed in a study by Fraser *et al.* [16] using a different MLST scheme, 24 were from Australia. Of these 24 Australian isolates, 50% (n = 12) showed an identical allelic profile, corresponding to the VGIIb genotype. Thus, their results are consistent with our findings of a low-level genetic diversity and an asexual reproduction structure of the Australian *C. gattii* VGII population. Such a low genetic diversity is supported by comparisons with other regions, especially South America, where of the seven MLST loci investigated, each observed sequence type corresponded to a distinct genotype (Table S1).

ST7 is numerically abundant and geographically widespread in Australia. Indeed, it was present in all four different regions investigated, *i.e.* QLD, NSW, NT and WA. Furthermore, it was the preponderant sequence type isolated. These large numerical and spatial representations of ST7 suggest that this genotype harbours intrinsic abilities to survive and flourish under different environmental conditions, from a tropical climate in Arnhemland at the “top-end” of Australia to much more temperate climates in the Sydney area of NSW and Perth in WA [32]. It thus could be categorized as a generalist genotype with the capacity to colonize various habitats. This observation is further reinforced by the detection of this genotype in other parts of the world (Table S1) [16], [27], [33]. In contrast to the widespread genotype ST7, five other sequence types encountered showed a more restricted spatial distribution. ST5, ST38 and ST48 were only found in WA, while the ST21 was found only in NSW. ST33 was mostly present in the NT, although, one isolate of this MLST type was also isolated from WA (Figure 4). This raises the question whether these sequence types are largely endemic to a restricted geographical region, and as such, are they more ecologically specialized compared to the more cosmopolitan ST7?

The occurrence of several MLST types throughout Australia, especially of ST7, is a striking finding and may indicate the ability of *C. gattii* VGII to disperse over long geographical distances. A previous study, investigating the genetic diversity and the associated population structure using AFLP markers on VGII isolates from the NT and the Sydney area, revealed a genetic differentiation between both defined populations [25]. However, when the authors restricted the analyses to closely related samples, a decreased statistical significance of the test of genetic differentiation was observed, leading the authors to conclude that there are potential genetic exchanges over a large spatial range [25]. This notion of long-distance dispersal within the *C. gattii* VGII population is somehow supported by the detection of the sequence type ST20 (VGIIa) from European patients, which had previously travelled to Vancouver Island and those had physically transposed this genotype to Europe (Table S1) [34], [35]. Several examples for the occurrence of long-distance dispersal events in the fungal kingdom have been well documented; particularly among plant pathogenic fungi [36]. Dissemination of fungi might be due to various vectors, for instance, transport of infected plant material [16], airborne dispersal [36], [37] and/or animal activities, such as migrating birds [37]. Dispersal of asexual spores and/or yeast cells of ST7 by different and undetermined biological and/or mechanical vectors might account for its abundance and widespread distribution throughout the Australian continent, and indeed, around the planet.

Sexual recombination has been suggested as a major force for the natural evolution of virulence [19]. For *C. gattii* VGII, same-sex mating between α-partners has been suggested, as the mechanism underlying the emergence of the Vancouver Island outbreak in British Columbia (Canada) based largely on circumstantial evidence [16], [30]. One of the scenarios postulated is that the hypervirulent VGIIa genotype (ST20 in the current study) originated from a mating event between two *MAT*α parents, namely the low virulent genotype VGIIb (ST7 in the current study) and an unknown mating partner. This speculation draws on the observation that, to date, only α-isolates have been detected on Vancouver Island and the notion that recombination has been detected within Australian populations constituted exclusively of VGII *MATα* - isolates [25]. Indeed, prior to the present study, *MAT* **a** isolates had never been detected in Australia [16], [25]. As suggested by Hiremath *et al.* [38], *MAT* **a** strains may contribute critically to breeding, but are in such low overall abundance that they are difficult to isolate via routine environmental sampling.

Population genetic studies generally require two main aspects, (i) the use of polymorphic molecular markers, such as the seven MLST-ISHAM-adopted loci and (ii) access to a sampling size as large as possible. The extensive molecular analyses realized in this study have demonstrated for the first time the presence of the mating type **a** in Australia, more precisely in south-western WA. Two isolates belonging to the same MLST genotype (ST38) were characterized as *MAT* **a**. One isolate was isolated from a veterinary case, a Dalmatian dog from the Geraldton area, WA, in 2001, and the second one from a eucalyptus tree trunk (species not determined) from the Caversham Wildlife Park to the north of Perth, WA, in 2009, approximately 400 km apart. This clearly indicates that *MAT* **a**-strains are present in the Australian environment. The current study further suggests sexual recombination among VGII *MAT*α and *MAT* **a** strains in Australia, an observation in accordance with the natural life cycle of *C. gattii*. Sexuality among α- and **a**-mating partners has also been suggested in the related *C. gattii* molecular type VGI [12], [39].

In this context, it is important to note that when a pattern of recombination is detected, it is hard to differentiate whether it corresponds to a past or a contemporary event [31]. Regarding the Vancouver Island outbreak, same-sex mating could be the driving force, but alternative processes might be involved, such as long distance dispersal events and multiple introduction phenomena. To answer this question, further population genetic investigations based on an extensive sampling on a global scale are currently underway by our research team using the international ISHAM consensus MLST scheme.

The findings presented here suggest that asexual reproduction and sexual recombination both contribute to the genetic diversity and structure of the Australian *C. gattii* VGII population. Clonality and sexual reproduction are not mutually exclusive. Evidence of both modes has already been highlighted [38]. The relative importance of the breeding system, *i.e.* asexual *versus* sexual reproduction, has important evolutionary implications. Asexual reproduction promotes the colonization of new habitats and infections by one or a few clones [20], whereas sexual reproduction favours genetic re-assortment with increased probability of survival in changing and/or competitive environments [18]. Indeed, it has been shown in Thailand that genotypes of *Penicillium marneffei*, an opportunistic fungus capable of infecting HIV/AIDS patients, may be clustered according to ecological conditions. This suggests that clonality has led to the evolution of niche-adapted genotypes [20]. Based on experiments in the laboratory using populations of the yeast *Saccharomyces cerevisiae*, Goddard *et al.* [18] demonstrated that sex can provide a selective advantage for adaptation to new environmental conditions. Combination of both modes – sexual and asexual – could greatly facilitate the population expansion of microorganisms.

Mismatch distributions for four of the seven loci were consistent with the distribution obtained under the sudden expansion model and thus may indicate an historical population expansion. Furthermore, repartitions of pairwise differences were typically L-shaped, a pattern consistent with a bottleneck phenomenon followed by a demographic expansion [40]. A possible scenario would involve the Australian *C. gattii* VGII population having undergone a reduction of its population size resulting in a historic founder effect (colonization of a new habitat by few individuals, in this case yeast cells or spores). This presumptive founder effect was subsequently followed by asexual population growth. It has been postulated that *C. gattii* originating from Australia was subsequently exported to other regions of the world by the transport of eucalyptus trees [10]. This hypothesis is to some extent, supported by Fraser and colleagues [16], who argue that Australia is the source of the Vancouver Island outbreak through an introduction of the VGIIb genotype to North West America. In contradiction, the present results of low genetic diversity, clonal structure and founder effect taken together suggest an alternative hypothesis, that the molecular type VGII has been introduced to Australia in the past, while persisting in its natural environment within other geographical regions.

Considering the behaviour of emerging and re-emerging infectious diseases [29], [30], [41], the ST7 (VGIIb), that is widely dispersed around Australia and accounts for numerous human and animal cases, could potentially be responsible for triggering an ongoing outbreak on a continental scale in Australia. It could be argued that, together with ST33, it already is responsible for an outbreak in Arnhemland, NT, whose scale is diminished only by the low population density of indigenous aboriginals in this location. The incidence rate of cryptococcosis in Arnhemland certainly rivals the one of Vancouver Island, Canada [42]. Although isolates of VGIIb (ST7) have been characterized as being of low virulence when compared to genotype VGIIa (ST20) in mice models [16], [43], a retrospective survey from 1999 to 2007 from Vancouver Island revealed that human death is actually more likely to be attributable to VGIIb infections. Furthermore, VGIIa infections apparently do not cause more severe illness than those caused by VGIIb strains [44]. According to our records, the first human infections attributed to *C. gattii* VGII occurred in 1983 and 1985, in the NT and WA, respectively (Table S1). The first veterinary evidence of VGII infection was reported from a horse in 1988 (Table S1) [9]. Recently, it has been proposed that canine and feline cryptococcosis due to VGII isolates may be increasing in WA [8]. This is further supported by the detection of an outbreak affecting simultaneously over 100 sheep near Busselton, WA, that was investigated in 1993 (Table S1) [24]. In addition, Caversham Wildlife Park continues to have a very high environmental presence of VGII (including isolates of several different sequence types types, one being of the **a** mating type), with a high prevalence of asymptomatic nasal colonisation, subclinical infection and clinical disease in exhibited animals (koalas and wombats), which persists despite attempted environmental control measures (Mark Krockenberger and Karen Payne, personal communication). Overall, these observations are consistent with the potential for more widespread outbreaks due to *C. gattii* VGII strains in Australia, in either south-western WA and/or Arnhemland, NT. Despite the fact that this study contains the largest set of VGII isolates ever collected from Australia, the limited sample size can only point to the possibility of an outbreak at a continental scale in Australia. However, the existing data without doubt emphasizes the need for an on-going surveillance of environmental, clinical and veterinary cryptococcal isolates from Australia to identify the extent of clonal outbreaks that might account for cases in high incidence areas. A problem concerns the question of how to monitor regions where the population density of humans and domesticated animals is low [26], [45]–[47].

A secondary outcome of the current study has been the demonstration that MLST genotyping results in stable, robust and reproducible data [48], which permits comparisons between different research groups and an exchange of typing data via web-based databases (*e.g.* MLST home page: http://www.mlst.net). As a result of the current study an online database for *C. gattii* has been established (http://mlst.mycologylab.org) on the basis of the seven loci adopted by the ISHAM Working group for genotyping of *C. neofromans* and *C. gattii* [48]. The database enables online single or multiple loci assignments using polyphasic sequence alignment algorithms. In addition the database allows online depositing of interesting strains, associated data and sequences, allowing the cryptococcal research community to contribute to a better understanding of the global *C. gattii* population diversity. Considering intra-MLST comparisons, this study demonstrated unambiguously that the seven chosen loci were sufficient to analyze the genetic variability within the Australian *C. gattii* population, with the genotypic diversity reaching a plateau for a total of only three loci (Figure 2).

### Conclusion

The investigation of the molecular epidemiology of *C. gattii* VGII on a large geographical scale in Australia has led to two key findings. Firstly, the presence of both *MAT* **a** and *MAT*α strains and the detection of potential recombination suggest the presence of sexual breeding between opposite mating types. Secondly, the data have revealed evidence of a potential on-going outbreak throughout Australia due to a limited number of VGII genotypes, possibly caused by a founder effect followed by clonal expansion. Finally, to understand the underlying mechanisms of fungal emergence and spread in Australia, Vancouver Island and elsewhere, a global population genetics approach using the internationally adopted MLST scheme and web-based databases is required.

## Materials and Methods

### VGII C. gattii isolates studied

Fifty-four Australian *C. gattii* VGII isolates were retrieved from the Molecular Mycology Research Laboratory culture collection (Westmead Hospital, University of Sydney, Westmead, NSW, Australia) (Table S1), representing the major areas from which *C. gattii* VGII has been isolated (2 from QLD, 10 from NSW, 10 from NT and 32 from WA). These isolates reflect also all possible isolation sources (14 clinical, 28 veterinary and 12 environmental) (Table S1).

The strains CDC R265 (representing the VGIIa, major Vancouver Island outbreak genotype) and CDC R272 (representing the VGIIb, minor Vancouver Island genotype) [16], [27] were included as reference strains (Table 1 and Table S1). To place the Australian VGII population in the context of the worldwide population, 6 isolates from North America, 3 from Europe, 1 from Asia and 7 from South America were also included (Table S1). Therefore a total of 71 *C. gattii* VGII isolates were studied.

### DNA extraction

Isolates were subcultured onto Sabouraud Dextrose Agar (SDA) at 37°C for 72 h prior to DNA extraction. High molecular weight DNA was than extracted according to Ferrer *et al.* [49] with minor modifications. Half an inoculation loop of the culture was transferred to a microcentrifuge tube and kept at −20°C overnight. Thereafter, the fungal material was incubated at 65°C for 1 h with 500 µl of lysis buffer (17.3 mM SDS, 0.25 M NaCl, 25 mM EDTA, 0.2 M Tris-HCl) and 5 µl of 2-mercaptoethanol. After incubation, 500 µl of phenol-chloroform-isoamyl alcohol (25∶24∶1), vol/vol/vol) were added to the tube and the mixture centrifuged at 14,000 rpm for 15 min. The upper phase was taken and mixed with an equal volume of isopropanol and the DNA was precipitated at −20°C overnight. After washing with 70% ethanol, the DNA pellet was resuspended in sterile deionized water. DNA concentration was determined by reading the UV absorbance at 260 nm (BioPhotometer, Eppendorf) and diluted to 10 ng/µl.

### Molecular typing

Restriction fragment length polymorphism (RFLP) analysis of the *URA5* gene via double digestion with the enzymes *Hha*I and *Sau*96I was performed to determine the molecular types, as previously described [22].

### Mating type identification

To determine the mating type of all studied isolates, a mating type specific polymerase chain reaction (PCR) was carried out using the α mating type specific primer pair MFαU and MFαL [12], and the **a** mating type specific primer pair JOHE9787 and JOHE9788 [50] (Table S2). Amplifications were performed as previously published [12], [50]. PCR reactions were repeated independently, three times, for the two samples identified as mating type **a** (see Results).

### Multilocus sequence typing (MLST)

The genetic variation within the Australian VGII population was studied using the ISHAM consensus MLST scheme for the *C. neoformans*/*C. gattii* species complex [48]. The typing scheme consists of seven unlinked genetic loci, including six housekeeping genes, namely the capsular associated protein (*CAP59*), glyceraldehydes-3-phosphate dehydrogenase (*GPD1*), laccase (*LAC1*), phospholipase B (*PLB1*), Cu, Zn superoxide dismutase (*SOD1*) and orotidine monophosphate pyrophosphorylase (*URA5*), and a non-coding region, the intergenic spacer region of the rDNA (IGS1).

Amplifications were carried out in a 50 µl reaction volume, containing: 100 ng of template DNA, 0.2 mM of deoxynucleoside triphosphate each, 7.5 pmol of the appropriate primers [48] (Table S2), 2 mM of MgCl_2_, 2.5 U of taq polymerase (BIOTAQ™ DNA polymerase, BIOLINE), together with the buffer recommended by the manufacturer (10x NH_4_ Buffer, BIOLINE), following the published amplification conditions [48]. Purified PCR products were sent to MACROGEN (Seoul, Korea) for commercial sequencing. Sequences were edited using Sequencer version 4.7 (Gene Codes, Ann Arbor, MI).

### Genetic variability

Each sequence was assigned a unique MLST allele number. Allele numbers were assigned for the following five loci: *CAP59*, *GPD1*, *PLB1*, *LAC1* and IGS1, according to Fraser *et al.* [16] and Byrnes *et al.* [29], [30]. For each new allele identified, a new allele number was given in order of discovery. For the *URA5* and *SOD1* loci, allele identification was undertaken by comparison with our own global cryptococcal sequence database. Due to the lack of a *C. gattii* MLST database, an MLST database based on BioloMICS software (BioAware, Belgium) was constructed for the 7 ISHAM consensus loci at the Molecular Mycology Research Laboratory and can be accessed at http://mlst.mycologylab.org. For allele identification, sequences were aligned using CLUSTAL X version 2.0 [51]. GenBank accession numbers for all MLST sequences used in this study are listed in Table S3. The allele numbers of the 7 genetic loci sequenced gave allelic profiles and allowed the designation of Sequence Types (STs). For example, the strain CDC R272 presents the following profile: *CAP59*-2, *GPD1*-6, *LAC1*-4, *PLB1*-2, *SOD1*-15, *URA5*-2, IGS1-10, which corresponds to the sequence type 7 (ST7). Each discrete sequence type was also assigned an arbitrary number in the order of detection. The sequence type numbers were given in accordance with a global study currently undertaken in our laboratory.

An unrooted Neighbor-Joining tree was constructed from the concatenated DNA sequences (a combination of the sequences from the 7 loci) of all Australian isolates using MEGA version 4 [52]. The genetic distance between isolates was computed using the *p*-distance and all positions containing alignment gaps were eliminated in the pairwise sequence comparisons. The significance of nodes was tested by bootstrapping with 1000 replications. The two strains CDC R265 and CDC R272 were included as reference strains in this analysis. An unrooted Neighbor-Joining tree has also been constructed considering all the sequence types delineated in Australia and the representative sequence types from other parts of the world. The isolates used to represent sequence types from other regions then Australia were selected for sequencing according to previous studies [16], [29], [30].

Nucleotide polymorphism positions for the 7 loci were determined using the software DnaSP version 5 [53] and checked manually with CLUSTAL X version 2.0 [51]. Polymorphic positions were obtained after alignment of the 54 Australian isolates with the two references strains CDC R265 (VGIIa) and CDC R272 (VGIIb). Gaps variations were not considered.

To determine whether the number of loci used was sufficient to access the genetic diversity in the Australian *C. gattii* VGII population we plotted genotypic diversity against the number of loci using Multilocus version 1.3 software [54]. Genotypic diversity is given as *n*/*n* – 1(1 – Σ*pi* ^2^) where *n* is the total number of individuals sampled and *pi* the relative frequency of the *i*th genotype [54]. The standard error was determined by 1000 randomizations.

### Tests for multilocus linkage disequilibrium

To test for multilocus linkage disequilibrium (*i.e.* non random association) among the 7 MLST loci the index of association *I_A_* and the slightly modified statistic *rBard* were computed using the Multilocus version 1.3 software [54]. Calculation of the *rBard* statistic has been performed in order to complement the *I_A_* index. Indeed, the *I_A_* value obtained is dependent of the number of loci included in the analyses, whereas *rBard* removes this dependency and would allow comparisons among studies [54]. The observed dataset is compared to 1000 datasets in which alleles have been randomly shuffled across isolates for each locus separately. The 1000 artificially produced datasets will thus simulate complete panmixia, *i.e.* infinite recombination. Thus, the null hypothesis of no linkage disequilibrium, consequently of recombination, will not be rejected if the observed values of both statistics are not significantly different from the distribution of the values obtained with the 1000 artificially recombining datasets. *I_A_* and *rBard* indexes were computed on (i) the complete dataset and (ii) the clone-corrected dataset from which replicates from the same sequence type were removed. Both complete and clone-corrected analyses were carried out on populations having a number of sequence types greater than 3, and consequently on the global and the WA populations.

### Demographic history

Historical demography of the Australian population was examined using two approaches. First, the distribution of the pairwise sequence differences, called mismatch distribution, [40], [55], [56] was generated for each locus and compared to the expected distribution under the sudden expansion model using Arlequin version 3.11 [57]. Goodness-of-fit between the observed and the expected mismatch distribution was tested using the sum of square deviation (SSD) approach. The 90% confidence interval of the expected mismatch distribution was also computed. Second, several statistical neutrality tests were used, including Tajima's *D* [58], Fu & Li's F* [59] and Fu's *F_S_* [60] statistics. These tests were computed independently for each locus using DnaSP version 5 [53]. Departure from the null hypothesis of neutral selection and/or constant population size was determined by generating 1000 permutations.

## Supporting Information

Table S1 List of isolates used in this study and their related information: location, source of isolation (CLIN: clinical, VET: veterinary and ENV: environmental), specific source and date of isolation. Mating types, allele numbers for the seven MLST loci and the corresponding sequence type (ST) are also presented.
(DOC)

Table S2 List of primers used in this study.
(DOC)

Table S3 GenBank accession numbers for the alleles obtained in the current study of the seven MLST loci studied.
(DOC)
