# Supplementary material for: Clonality and α-a Recombination in the Australian Cryptococcus gattii VGII Population - An Emerging Outbreak in Australia
Source: PLoS One. 2011 Feb 24;6(2):e16936. doi: 10.1371/journal.pone.0016936 (PMC3044715; doi:10.1371/journal.pone.0016936)
Supplement: Table S1 — List of isolates used in this study and their related information: location, source of isolation (CLIN: clinical, VET: veterinary and ENV: environmental), specific source and date of isolation. Mating types, allele numbers for the seven MLST loci and the corresponding sequence type (ST) are also presented. (DOC) [file pone.0016936.s001.doc]

**Table S1:** List of isolates used in this study and their related information: location, source of isolation (CLIN: clinical, VET: veterinary and ENV: environmental), specific source and date of isolation. Mating types, allele numbers for the seven MLST loci and the corresponding sequence type (ST) are also presented.

|  | **WM number** | **Other collection number** | **Specific location** | **Source of isolation** | **Specific source** | **Isolation date** | **Mating type** | ***CAP59*** | ***GPD1*** | ***LAC1*** | ***PLB1*** | ***SOD1*** | ***URA5*** | **IGS1** | **ST** |
| --- | --- | --- | --- | --- | --- | --- | --- | --- | --- | --- | --- | --- | --- | --- | --- |
| Australia | WM 1008 |  | Australia, NSW, Blacktown | ENV | *Eucalyptus tereticornis*, association with frass of a moth larva | 2000 | α | 2 | 6 | 4 | 2 | 15 | 2 | 10 | **7** |
|  | WM 437 | New8; H 18 | Australia, NSW, Newcastle | CLIN | Skin | 1994 | α | 2 | 6 | 4 | 2 | 15 | 2 | 10 | **7** |
|  | WM 198 | VPB 571-015; McBride | Australia, NSW, Sydney, Camden | VET | Cat | 1990 | α | 2 | 6 | 4 | 2 | 15 | 2 | 10 | **7** |
|  | WM 08.245 | 571 270 | Australia, NSW, Sydney | VET | Cat (15 years old), lymph node | 1996 | α | 2 | 6 | 4 | 2 | 15 | 2 | 10 | **7** |
|  | WM 178^**,¶^ | 49435; CBS 10082; IFM 50894 | Australia, NSW, Sydney | CLIN | Lung | 1991 | α | 1 | 17 | 16 | 14 | 19 | 7 | 16 | **21** |
|  | WM 05.230^‡,¶^ | 571116; V20 | Australia, NSW, Sydney, Bradbury | VET | Dog, abdominal mass | 1995 | α | 2 | 6 | 4 | 2 | 15 | 2 | 10 | **7** |
|  | WM 04.71^†,‡,¶^ | VPB 571-058; PR563; V4; CP 2500 / 91 | Australia, NSW, Sydney, Chiswick | VET | Cat (Birman cat), nasal | 1991 | α | 2 | 6 | 4 | 2 | 15 | 2 | 10 | **7** |
|  | WM 08.284^†,‡,¶^ | C 194; V9 | Australia, NSW, Sydney, Coogee | VET | Cat (Pusska Shepherd) | 1992 | α | 2 | 6 | 4 | 2 | 15 | 2 | 10 | **7** |
|  | WM 08.267 | 571 327 | Australia, NSW, Sydney, Gladesville | VET | Cat, nasal | 2006 | α | 2 | 6 | 4 | 2 | 15 | 2 | 10 | **7** |
|  | WM 2209 | 1607/96 | Australia, NSW, Sydney, Rosemeadow | VET | Cat (3.5 years old), nasal | 1996 | α | 2 | 6 | 4 | 2 | 15 | 2 | 10 | **7** |
|  | WM 04.70^†,‡,¶^ | RDH06 | Australia, NT, Arnhemland | CLIN | CSF | 1996 | α | 2 | 6 | 4 | 2 | 15 | 2 | 10 | **7** |
|  | WM 06.1^**,†,‡,¶^ | NT-08 | Australia, NT, Arnhemland | CLIN | Lung | 1983 | α | 3 | 2 | 7 | 1 | 18 | 5 | 6 | **33** |
|  | WM 2606^†,‡^ | H 16 | Australia, NT, Arnhemland | CLIN | Lung | 1996 | α | 3 | 2 | 7 | 1 | 18 | 5 | 6 | **33** |
|  | WM 3030^†,‡,¶^ | NT-07 | Australia, NT, Arnhemland | CLIN | CSF | 1989 | α | 3 | 2 | 7 | 1 | 18 | 5 | 6 | **33** |
|  | WM 03.456^¶^ | ARN 001 | Australia, NT, Arnhemland, | ENV | *Eucalyptus* tree (near house) | 21/06/1999 | α | 2 | 6 | 4 | 2 | 15 | 2 | 10 | **7** |
|  | WM 05.229^**,†,‡,¶^ | NT-13 | Australia, NT, Arnhemland | CLIN | Lung | 1985 | α | 2 | 6 | 4 | 2 | 15 | 2 | 10 | **7** |
|  | WM 03.27^¶^ | RAM 002 | Australia, NT, Arnhemland | ENV | *Eucalyptus* tree | 22/06/1999 | α | 2 | 6 | 4 | 2 | 15 | 2 | 10 | **7** |
|  | WM 04.187^‡,¶^ | RAM 005 | Australia, NT, Arnhemland | ENV | *E. tetrodonta* | 22/06/1999 | α | 2 | 6 | 4 | 2 | 15 | 2 | 10 | **7** |
|  | WM 04.69^‡,¶^ | RAM 015 | Australia, NT, Arnhemland | ENV | *E. miniata* (100 m north of oval) | 22/06/1999 | α | 2 | 6 | 4 | 2 | 15 | 2 | 10 | **7** |
|  | WM 3032^†,‡^ | NT-14 | Australia, NT, Katherine Region | CLIN | CSF | 1983 | α | 3 | 2 | 7 | 1 | 18 | 5 | 6 | **33** |
|  | WM 09.4 | 37098-9217 | Australia, QLD, Aurukun | CLIN | Male (50 years old) | 2008 | α | 2 | 6 | 4 | 2 | 15 | 2 | 10 | **7** |
|  | WM 08.141 | 571156 | Australia, QLD, Maleny | VET | Cat, nasal | 1996 | α | 2 | 6 | 4 | 2 | 15 | 2 | 10 | **7** |
|  | WM 08.306 | PWQ 875, 5018; Q99; 86773K | Australia, WA | VET | Horse (name RIjInski ) | 15/12/1999 | α | 2 | 6 | 4 | 2 | 15 | 2 | 10 | **7** |
|  | WM 08.308; WM 2971 | PWQ 606, 14-1419; S00 3895-U; MA 21832 | Australia, WA, Perth | VET | Horse (name: Olivieri; 5 years old), tracheal wash | 12/07/1995 | α | 2 | 6 | 4 | 2 | 15 | 2 | 10 | **7** |
|  | WM 09.85 | PWQ 320; 91/684; 91/183; | Australia, WA | VET | Horse (name: Grey all noble), tracheal wash | 06/02/1991 | α | 2 | 6 | 4 | 2 | 15 | 2 | 10 | **7** |
|  | WM 09.86 | PWQ 321; 88/1337; 88/460 | Australia, WA | VET | Horse (cough after exercise, lung lesion) | 08/03/1988 | α | 2 | 6 | 4 | 2 | 15 | 2 | 10 | **7** |
|  | WM 09.88 | PWQ 494; 93/3258 | Australia, WA, near Busselton | VET | Sheep L2 (horn lesion) | 04/06/1993 | α | 2 | 6 | 4 | 2 | 15 | 2 | 10 | **7** |
|  | WM 09.89 | PWQ 496; 93/3257 | Australia, WA. near Busselton | VET | Sheep L1 (eye lesion) | 04/06/1993 | α | 2 | 6 | 4 | 2 | 15 | 2 | 10 | **7** |
|  | WM 09.90; WM 08.307 | PWQ 474; P93/133; 93/355; 14-1408 | Australia, WA, near Busselton | VET | Sheep 093-133, cid (tumor) | 20/01/1993 | α | 2 | 6 | 4 | 2 | 15 | 2 | 10 | **7** |
|  | WM 09.91 | PWQ 628; 95/3662 | Australia, WA | VET | Horse (name: Gavin "Kiwi Habit") tracheal wash | 24/05/1995 | α | 2 | 6 | 4 | 2 | 15 | 2 | 10 | **7** |
|  | WM 09.98 | PWQ 1781; P08/2061902G | Australia, WA | VET | Dog, cid, nasal swab | 16/12/2008 | α | 2 | 6 | 4 | 2 | 15 | 2 | 10 | **7** |
|  | WM 09.94 | PWQ 1009; Q01/1067864N | Australia, WA, Geraldton area | VET | Dog, cid, cerebrospinal fluid (3 years old) | 21/12/2001 | **a** | 14 | 21 | 28 | 27 | 46 | 2 | 27 | **38** |
|  | WM 08.309; WM 447 | PWQ 730, 14-1431; SOLIDAGO; M31557; M29004 | Australia, WA | VET | Horse (name: Solidago), lung | 04/04/1997 | α | 27 | 6 | 4 | 1 | 43 | 7 | 4 | **48** |
|  | WM 08.310 | PWQ 655, 14-1433; Malley; M25183 | Australia, WA | VET | Horse (name: Malley Natasi Aztec) | 29/11/1995 | α | 27 | 6 | 4 | 1 | 43 | 7 | 4 | **48** |
|  | WM 09.97 | PWQ 1675; P08/2030588F | Australia, WA, Busselton, Alton Park | VET | Cow # 1004 (tumor-like lesions through the lungs) | 11/03/2008 | α | 2 | 6 | 4 | 2 | 15 | 2 | 10 | **7** |
|  | WM 09.153 | - | Australia, WA, Caversham Wildlife Park | ENV | Eucalyptus log under Koala, new shed | 08/07/2009 | α | 2 | 6 | 4 | 2 | 15 | 2 | 10 | **7** |
|  | WM 09.154 | - | Australia, WA, Caversham Wildlife Park | ENV | Eucalyptus log, new shed | 08/07/2009 | α | 2 | 6 | 4 | 2 | 15 | 2 | 10 | **7** |
|  | WM 09.163 | - | Australia, WA, Caversham Wildlife Park | ENV | Eucalyptus tree trunk I, old shed | 08/07/2009 | α | 2 | 6 | 4 | 2 | 15 | 2 | 10 | **7** |
|  | WM 09.164 | - | Australia, WA, Caversham Wildlife Park | ENV | Vertical Eucalyptus log, old shed | 08/07/2009 | α | 2 | 6 | 4 | 2 | 15 | 2 | 10 | **7** |
|  | WM 09.166 | - | Australia, WA, Caversham Wildlife Park | ENV | Vertical Eucalyptus log (second), visitor area | 08/07/2009 | α | 2 | 6 | 4 | 2 | 15 | 2 | 10 | **7** |
|  | WM 09.155 | - | Australia, WA, Caversham Wildlife Park | VET | Koala (name: Sarah), nasal swab right nose^●^, new shed | 08/07/2009 | α | 2 | 6 | 4 | 2 | 15 | 2 | 10 | **7** |
|  | WM 09.156 | - | Australia, WA, Caversham Wildlife Park | VET | Koala (name: J), nasal swab right nose^●●^, new shed | 08/07/2009 | α | 2 | 6 | 4 | 2 | 15 | 2 | 10 | **7** |
|  | WM 09.157 | - | Australia, WA, Caversham Wildlife Park | VET | Koala (name: Amie), nasal swab right nose^●^, new shed | 08/07/2009 | α | 2 | 6 | 4 | 2 | 15 | 2 | 10 | **7** |
|  | WM 09.158 | - | Australia, WA, Caversham Wildlife Park | VET | Koala (name: Amelie), nasal swab left nose^●^, new shed | 08/07/2009 | α | 2 | 6 | 4 | 2 | 15 | 2 | 10 | **7** |
|  | WM 09.160 | - | Australia, WA, Caversham Wildlife Park | VET | Koala (adult male), nasal swab left and right nose^●^, new shed | 08/07/2009 | α | 2 | 6 | 4 | 2 | 15 | 2 | 10 | **7** |
|  | WM 09.161 | - | Australia, WA, Caversham Wildlife Park | VET | Koala (no name, female), nasal swab left and right nose^●^, new shed | 08/07/2009 | α | 2 | 6 | 4 | 2 | 15 | 2 | 10 | **7** |
|  | WM 09.165 | - | Australia, WA, Caversham Wildlife Park | ENV | Eucalyptus tree trunk II, old shed | 08/07/2009 | **a** | 14 | 21 | 28 | 27 | 46 | 2 | 27 | **38** |
|  | WM 09.152 | - | Australia, WA, Caversham Wildlife Park | ENV | Eucalyptus log above Eucalyptus trunk, new shed | 08/07/2009 | α | 27 | 6 | 4 | 1 | 43 | 7 | 4 | **48** |
|  | WM 09.83 | PWQ 309 | Australia, WA, Mandurah | CLIN | Male (54 years old) cerebrospinal fluid | 17/12/1985 | α | 27 | 6 | 4 | 1 | 43 | 7 | 4 | **48** |
|  | WM 09.95 | PWQ 1040 | Australia, WA, Meekatharra | CLIN | Female (24 years old) Sputum | 26/08/2002 | α | 27 | 6 | 4 | 1 | 43 | 7 | 4 | **48** |
|  | WM 09.96 | PWQ 1671 | Australia, WA, Middle Swan | CLIN | Male (40 years old) cerebrospinal fluid | 29/01/2008 | α | 27 | 6 | 4 | 1 | 43 | 7 | 4 | **48** |
|  | WM 08.311; WM 08.131; WM 2517^*,**,¶^ | PWQ 697, 14-1434; Jackson; 6003508t; 571140; WA861 | Australia, WA, Perth | VET | Dog (Dalmatian), cranial mediastinal mass | 10/04/1996 | α | 3 | 16 | 4 | 9 | 23 | 2 | 15 | **5** |
|  | WM 1382 | PWQ 777 | Australia, WA, Perth, Willetton | CLIN | Male (40 years old), cerebrospinal fluid | 18/03/1998 | α | 2 | 6 | 4 | 2 | 15 | 2 | 10 | **7** |
|  | WM 04.72 | PWQ 314; W12 | Australia, WA, Perth | CLIN | Male (49 years old) | 31/07/1990 | α | 3 | 2 | 7 | 1 | 18 | 5 | 6 | **33** |
| North America | WM 02.32^*,**,‡‡^ | CDC R265 | Canada, British Columbia, Vancouver Island, Duncan | CLIN | Male, bronchial wash | 2001 | α | 1 | 1 | 4 | 1 | 14 | 7 | 4 | **20 (=VGIIa)** |
|  | WM 06.25; WM 02.39^*,**,‡‡^ | CDC R272 | Canada, British Columbia, Vancouver Island, Ladysmith | CLIN | Female, bronchial wash | 2001 | α | 2 | 6 | 4 | 2 | 15 | 2 | 10 | **7 (=VGIIb)** |
|  | - | B7432 | USA, Oregon | CLIN | Blood | 05/01/2009 | - | 4 | 6 | 4 | 1 | 15 | 2 | 15 | **6 (=VGIIc)** |
|  | WM 06.4^¶,**^ | ATCC 32609.  NIH 444. CBS 6956 | USA, Maryland, Bethesda | CLIN | Sputum | 1975 | α | 1 | 1 | 4 | 1 | 14 | 7 | 4 | **20** |
|  | WM 06.13^¶,**^ | CBS 7750 ;  451-SF13.2 | USA, California, San Francisco | ENV | *E. camaldulensis* (bark debris) | 1990 | α | 1 | 1 | 4 | 1 | 14 | 7 | 4 | **20** |
|  | WM 02.35 | CDC R268 | Canada, British Columbia, Lantzville | CLIN | Female, cerebrospinal fluid | 2001 | α | 1 | 1 | 4 | 1 | 14 | 7 | 4 | **20** |
| Europe | WM 09.113 | CBS11511; RKI06/496 | Switzerland | CLIN | Female (53 years old), cerebrospinal fluid | 2006 | α | 1 | 1 | 4 | 1 | 14 | 7 | 4 | **20** |
|  | WM 09.144 | CBS10485; E758/2005 | Denmark, Herning | CLIN | Male (51 years old), lung biopsy | 2006 | α | 1 | 1 | 4 | 1 | 14 | 7 | 4 | **20** |
|  | WM 05.77** | AV55 ; CBS 10090 | Greece, Athens | CLIN | Female (26 years old) | 1998 | **a** | 2 | 27 | 4 | 1 | 8 | 7 | 10 | **18** |
| Asia | - | K71 | Republic of Korea, Gyeonggi | CLIN | Male (57 years old), cerebrospinal fluid | 2006 | α | 2 | 6 | 4 | 2 | 15 | 2 | 10 | **7** |
| South America | WM 06.33^¶,**^ | CBS 1930 ; RV 71421 | Aruba | VET | Goat | 1953 | **a** | 2 | 6 | 4 | 18 | 12 | 10 | 25 | **25** |
|  | WM 05.452** | LMM 417 ; LA 55 | Brazil, Piaui | CLIN | Male (18 years old), cerebrospinal fluid | 1995 | **a** | 2 | 27 | 4 | 1 | 8 | 7 | 15 | **16** |
|  | WM 05.470 | LMM 547.18.13A3 | Brazil, Piaui | ENV | Wood | 1994 | α | 2 | 16 | 4 | 26 | 15 | 2 | 42 | **26** |
|  | WM 06.8^¶,**^ | LA 43 ; NUAM 176; CBS 8684 | Uruguay, Quebrada de los Cuervos | ENV | Nest of wasp *Polybia occidentalis* | 1996 | α | 7 | 6 | 21 | 25 | 2 | 2 | 32 | **3** |
|  | WM 05.533 | LMM 892 | Brazil, Mato Grosso do Sul | CLIN | Female (18 years old), cerebrospinal fluid | 1997 | α | 10 | 6 | 30 | 16 | 20 | 2 | 16 | **11** |
|  | WM 477 | LA 84 ; HEC 11102 ; LMM 21 | Brazil, Rio de Janeiro | CLIN | Male (47 years old), urine | 1994 | α | 28 | 6 | 29 | 18 | 1 | 1 | 15 | **4** |
|  | WM 05.529 | LMM 855 | Brazil, Roraima | CLIN | Male (33 years old), cerebrospinal fluid | 1997 | α | 2 | 32 | 30 | 1 | 13 | 7 | 25 | **27** |

Isolates used in other population genetic studies: ^†^ [25]; ^‡^ [17]; ^‡‡^ [27]; ^¶^ [16]; * [29]; ** [30]; ^●^ Animals were colonized or had subclinical infections; ^●●^ Koala with an infection of the right claw treated with Fluconazole daily and Amphotericin B 2x weekly.
