# Supplementary material for: Clonality and α-a Recombination in the Australian Cryptococcus gattii VGII Population - An Emerging Outbreak in Australia
Source: PLoS One. 2011 Feb 24;6(2):e16936. doi: 10.1371/journal.pone.0016936 (PMC3044715; doi:10.1371/journal.pone.0016936)
Supplement: Table S2 — List of primers used in this study. (DOC) [file pone.0016936.s002.doc]

**Table S2:** List of primers used in this study.

| **Locus** | **Primer Sequence** | **Reference** |
| --- | --- | --- |
| **Pheromone *MFα*** (α Mating Type) | **MFαU** 5' TTCACTGCCATCTTCACCACC 3'  **MFαL** 5' TCTAGGCGATGACACAAAGGG 3' | 12 |
| **Pheromone *MF*a** (**a** Mating Type) | **JOHE9787** 5' ACACCGCCTGTTACAATGGAC 3'  **JOHE9788** 5' CAGCGTTTGAAGATGGACTTT 3' | 50 |
| ***CAP59*** (capsular associated protein) | **CAP59F** 5’ CTCTACGTCGAGCAAGTCAAG 3’  **CAP59R** 5’ TCCGCTGCACAAGTGATACCC 3’ | 48 |
| ***GPD1*** (glyceraldehydes-3-phosphate dehydrogenase) | **GPD1F** 5’ CCACCGAACCCTTCTAGGATA 3’  **GPD1R** 5’ CTTCTTGGCACCTCCCTTGAG 3’ | 48 |
| ***LAC1*** (laccase) | **LAC1F** 5’ AACATGTTCCCTGGGCCTGTG 3’  **LAC1R** 5’ ATGAGAATTGAATCGCCTTGT 3’ | 48 |
| ***PLB1*** (phospholipase B) | **PLB1F** 5’ CTTCAGGCGGAGAGAGGTTT 3’  **PLB1R** 5’ GATTTGGCGTTGGTTTCAGT 3’ | 48 |
| ***SOD1*** (Cu, Zn superoxide dismutase) | **SOD1CGF** 5’ GATCCTCACGCCATTACG 3’  **SOD1CGR** 5’ GAATGATGCGCTTAGTTGGA 3’ | 48 |
| ***URA5*** (orotidine monophosphate pyrophosphorylase) | **URA5F** 5’ ATGTCCTCCCAAGCCCTCGAC 3’  **URA5R** 5’ TTAAGACCTCTGAACACCGTACTC 3’ | 48 |
| **IGS1** (intergenic spacer region of the rDNA) | **IGSF** 5’ ATCCTTTGCAGACGACTTGA 3’  **IGSR** 5’ GTGATCAGTGCATTGCATGA 3’ | 48 |
