# Supplementary material for: Clonality and α-a Recombination in the Australian Cryptococcus gattii VGII Population - An Emerging Outbreak in Australia
Source: PLoS One. 2011 Feb 24;6(2):e16936. doi: 10.1371/journal.pone.0016936 (PMC3044715; doi:10.1371/journal.pone.0016936)
Supplement: Table S3 — GenBank accession numbers for the alleles obtained in the current study of the seven MLST loci studied. (DOC) [file pone.0016936.s003.doc]

**Table S3:** GenBank accession numbers for the alleles obtained in the current study of the seven MLST loci studied.

| **GenBank Accession number** | **Submission Details** | **References** |
| --- | --- | --- |
| FR677264 | MLST allele *CAP59_1* | 16 |
| FR677265 | MLST allele *CAP59_2* | 16 |
| FR677266 | MLST allele *CAP59_3* | 16 |
| FR677267 | MLST allele *CAP59_4* | 29 |
| FR677269 | MLST allele *CAP59_7* | 30 |
| FR677268 | MLST allele *CAP59_10* | This study |
| FR677270 | MLST allele *CAP59_14* | This study |
| FR677271 | MLST allele *CAP59_27* | This study |
| FR677272 | MLST allele *CAP59_28* | This study |
| FR677279 | MLST allele *GPD1_1* | 16 |
| FR677280 | MLST allele *GPD1_2* | 16 |
| FR677273 | MLST allele *GPD1_6* | 16 |
| FR677274 | MLST allele *GPD1_16* | 16 |
| FR677275 | MLST allele *GPD1_17* | 16 |
| FR677276 | MLST allele *GPD1_21* | 16 |
| FR677277 | MLST allele *GPD1_27* | 30 |
| FR677278 | MLST allele *GPD1_32* | This study |
| FR677281 | MLST allele *LAC1_4* | 16 |
| FR677282 | MLST allele *LAC1_7* | 16 |
| FR677283 | MLST allele *LAC1_16* | 16 |
| FR677284 | MLST allele *LAC1_21* | 16 |
| FR677285 | MLST allele *LAC1_28* | This study |
| FR677286 | MLST allele *LAC1_29* | This study |
| FR677287 | MLST allele *LAC1_30* | This study |
| FR677288 | MLST allele *PLB1_1* | 16 |
| FR677289 | MLST allele *PLB1_2* | 16 |
| FR677290 | MLST allele *PLB1_9* | 16 |
| FR677291 | MLST allele *PLB1_14* | 16 |
| FR677292 | MLST allele *PLB1_16* | 16 |
| FR677293 | MLST allele *PLB1_18* | 16 |
| FR677294 | MLST allele *PLB1_25* | This study |
| FR677295 | MLST allele *PLB1_26* | This study |
| FR677296 | MLST allele *PLB1_27* | This study |
| FR677297 | MLST allele *SOD1_1* | This study |
| FR677298 | MLST allele *SOD1_2* | This study |
| FR677299 | MLST allele *SOD1_8* | This study |
| FR677300 | MLST allele *SOD1_12* | This study |
| FR677301 | MLST allele *SOD1_13* | This study |
| FR677302 | MLST allele *SOD1_14* | This study |
| FR677303 | MLST allele *SOD1_15* | This study |
| FR677304 | MLST allele *SOD1_18* | This study |
| FR677305 | MLST allele *SOD1_19* | This study |
| FR677306 | MLST allele *SOD1_20* | This study |
| FR677307 | MLST allele *SOD1_23* | This study |
| FR677308 | MLST allele *SOD1_43* | This study |
| FR677309 | MLST allele *SOD1_46* | This study |
| FR677310 | MLST allele *URA5_1* | This study |
| FR677311 | MLST allele *URA5_2* | This study |
| FR677312 | MLST allele *URA5_5* | This study |
| FR677313 | MLST allele *URA5_7* | This study |
| FR677314 | MLST allele *URA5_10* | This study |
| FR677315 | MLST allele *IGS1_4* | 16 |
| FR677316 | MLST allele *IGS1_6* | 16 |
| FR677317 | MLST allele *IGS1_10* | 16 |
| FR677318 | MLST allele *IGS1_15* | 16 |
| FR677319 | MLST allele *IGS1_16* | 16 |
| FR677320 | MLST allele *IGS1_25* | 16 |
| FR677321 | MLST allele *IGS1_27* | 16 |
| FR677322 | MLST allele *IGS1_32* | This study |
| FR677323 | MLST allele *IGS1_42* | This study |
